# Prices and mark-ups on antimalarials: evidence from nationally representative studies in six malaria-endemic countries

**DOI:** 10.1093/heapol/czv031

**Published:** 2015-05-05

**Authors:** Benjamin Palafox, Edith Patouillard, Sarah Tougher, Catherine Goodman, Kara Hanson, Immo Kleinschmidt, Sergio Torres Rueda, Sabine Kiefer, Kate O’Connell, Cyprien Zinsou, Sochea Phok, Louis Akulayi, Ekundayo Arogundade, Peter Buyungo, Felton Mpasela, Stephen Poyer, Desmond Chavasse

**Affiliations:** ^1^London School of Hygiene and Tropical Medicine, Keppel Street, London WC1E 7HT, UK,; ^2^Swiss Tropical and Public Health Institute, Socinstrasse 57, 4051 Basel, Switzerland,; ^3^Population Services International, Malaria & Child Survival Department, PO Box 43640, Nairobi, Kenya,; ^4^Association Béninoise pour le Marketing Social/PSI, BP 08-0876, Tri Postal, Cotonou, Benin,; ^5^Population Services International Cambodia, No 29 St. 334, PO Box 153, BKK1 Chamcar Mon, Phnom Penh, Kingdom of Cambodia,; ^6^Association de Santé Familiale, 4630 Avenue de la Science, Immeuble USTC, Bloc C, Gombé, Kinshasa, Democratic Republic of Congo,; ^7^Society for Family Health, 8 Port Harcourt Crescent, Area 11 Garki, Abuja, Nigeria,; ^8^Programme for Accessible Health, Communication and Education, Plot 2 Ibis Vale, PO Box 27659, Kololo, Kampala, Uganda and; ^9^Society for Family Health, Plot No 549, Ridgeway, PO Box 50770, Lusaka, Zambia

**Keywords:** Antimalarials, malaria treatment, pharmaceutical pricing, private sector

## Abstract

The private for-profit sector is an important source of treatment for malaria. However, private patients face high prices for the recommended treatment for uncomplicated malaria, artemisinin combination therapies (ACTs), which makes them more likely to receive cheaper, less effective non-artemisinin therapies (nATs). This study seeks to better understand consumer antimalarial prices by documenting and exploring the pricing behaviour of retailers and wholesalers. Using data collected in 2009–10, we present survey estimates of antimalarial retail prices, and wholesale- and retail-level price mark-ups from six countries (Benin, Cambodia, the Democratic Republic of Congo, Nigeria, Uganda and Zambia), along with qualitative findings on factors affecting pricing decisions. Retail prices were lowest for nATs, followed by ACTs and artemisinin monotherapies (AMTs). Retailers applied the highest percentage mark-ups on nATs (range: 40% in Nigeria to 100% in Cambodia and Zambia), whereas mark-ups on ACTs (range: 22% in Nigeria to 71% in Zambia) and AMTs (range: 22% in Nigeria to 50% in Uganda) were similar in magnitude, but lower than those applied to nATs. Wholesale mark-ups were generally lower than those at retail level, and were similar across antimalarial categories in most countries. When setting prices wholesalers and retailers commonly considered supplier prices, prevailing market prices, product availability, product characteristics and the costs related to transporting goods, staff salaries and maintaining a property. Price discounts were regularly used to encourage sales and were sometimes used by wholesalers to reward long-term customers. Pricing constraints existed only in Benin where wholesaler and retailer mark-ups are regulated; however, unlicensed drug vendors based in open-air markets did not adhere to the pricing regime. These findings indicate that mark-ups on antimalarials are reasonable. Therefore, improving ACT affordability would be most readily achieved by interventions that reduce commodity prices for retailers, such as ACT subsidies, pooled purchasing mechanisms and cost-effective strategies to increase the distribution coverage area of wholesalers.

**Key Messages**

- Percentage mark-ups at retail and wholesale level were not excessive; although at retail-level mark-ups for artemisinin combination therapy (ACT) were lower than those applied to non-aretmisinin therapies, whereas wholesale-level mark-ups were more consistent across antimalarial type and lower than those at retail level.
- When determining antimalarial prices and mark-ups, wholesalers and retailers consider a range of factors related to operating expenses, competition, product availability, product characteristics and to a lesser degree price regulation and other pricing constraints.
- High private sector prices for ACT are an important barrier limiting access to effective treatment for malaria, and the findings of this study will be useful when developing interventions to improve the affordability of ACT.

## Introduction

In many low- and middle-income countries, the private for-profit sector is an important source of treatment for malaria, often in spite of free or highly subsidized treatment provision in the public sector. In some countries, private outlets are the initial source of treatment for nearly half of reported fevers (Littrell *et al*. 2011a,b) and distribute the majority of antimalarials (Littrell *et al*. 2011b; O'Connell *et al*. 2011). Given the high burden of malaria found in many countries, the private sector is therefore likely to have an important impact on health outcomes.

However, compared with those seeking treatment in the public sector, private patients are more likely to receive sub-optimal care: fewer private patients are diagnosed using either microscopy or rapid diagnostic test (RDT) prior to initiating treatment, and fewer receive the recommended treatment for uncomplicated *Plasmodium falciparum *(*P. falciparum*) malaria, artemisinin combination therapies (ACTs). Instead, private patients are more likely to receive older, less effective non-artemisinin therapies (nATs), such as chloroquine, quinine and sulphadoxine–pyrimethamine (Littrell *et al*. 2011a,b). In some countries, private retailers have also been found to distribute worrying amounts of artemisinin monotherapies (AMTs) in oral dosage forms (Littrell *et al*. 2011b; O'Connell *et al*. 2011; Tougher *et al*. 2012), potentially contributing to the spread of artemisinin resistance.

Understanding the private sector’s popularity and the persistent use of nATs by those who seek treatment there is complex. The choice of private over public providers is influenced by several factors (Rao *et al*. 2013). Private outlets are often closer to home; they may cost less overall; and compared with public facilities, they may be more likely to have antimalarials in stock when needed or perceived to offer better quality services. But when patients seek treatment in the private sector, they may encounter a wide range of provider options (O'Connell *et al*. 2011). Alongside private facilities staffed by medical doctors and nurses, and retail pharmacies supervised by registered pharmacists, malaria treatment may also be obtained from drug stores typically operated by those with little training, and a variety of unlicensed and unskilled retailers including grocery stores, kiosks, itinerant vendors and stalls in open-air markets. However, the types of private outlets accessible to patients vary by country and urban–rural location.

Private outlets may also offer consumers a bewildering array of antimalarial products including ACTs, AMTs and nATs; coming in tablet, oral liquid, granule, suppository and injectable dosage forms; as branded or unbranded; and as domestically manufactured or imported products. Despite this wide range of options, the type of antimalarial private patients come away with is strongly influenced by retail prices (Whitty *et al*. 2008; Alba *et al*. 2010). ACTs have been found to be many times more expensive than more popular alternatives (O'Connell *et al*. 2011). Consequently, high ACT prices are recognized as a key barrier to their wider use and contribute to maintaining demand for much cheaper nATs.

However, retailers are only the last link in a chain of businesses which includes manufacturers, importers and wholesalers, who are a diverse group of businesses themselves. For example, drug shops in Nigeria and open-air market stalls in Benin often act as wholesalers by supplying antimalarials to smaller retail outlets (Palafox *et al*. 2014). Therefore, understanding consumer prices for antimalarials not only requires insight into retailer characteristics, practices and pricing behaviour, but also into those of their supply sources. A 2010 review identified these areas as important knowledge gaps (Patouillard *et al*. 2010). The review also highlighted the lack of evidence on price mark-ups, particularly from wholesalers and unregistered suppliers; and on the factors influencing antimalarial prices.

The ACTwatch project was designed to address these evidence gaps by generating comprehensive data on antimalarial markets through linked studies of households, treatment sources and private sector distribution chains in several endemic countries (Shewchuk *et al*. 2011). Study countries were selected to represent a diverse range of contexts considering variation in malaria burden, the nature of pharmaceutical regulation (e.g. high vs low; francophone vs anglophone), public sector coverage and domestic antimalarial manufacturing capacity. Previous articles have presented cross-country evidence from ACTwatch on treatment seeking behaviour, the characteristics of retailers and the characteristics of wholesalers (Littrell *et al*. 2011a,b; O'Connell *et al*. 2011; Palafox *et al*. 2014). A range of reports provide more country-level data and analysis (Population Services International 2014).

**Figure 1.**
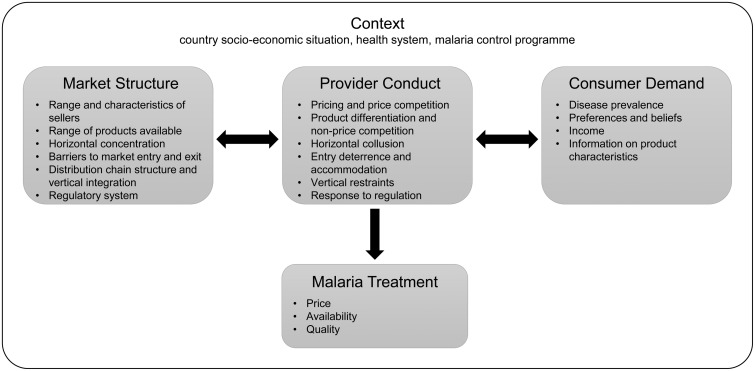
Structure, conduct, performance analytical framework (Goodman 2004; Patouillard 2012)

This article draws on the growing body of evidence from ACTwatch about the structure and operation of antimalarial distribution chains. It aims to document and explore the determinants of antimalarial retail prices in the private for-profit sectors of Benin, Cambodia, the Democratic Republic of Congo (DRC), Nigeria, Uganda and Zambia. To achieve this, we describe nationally representative estimates of antimalarial retail prices, and wholesale- and retail-level price mark-ups for each country, along with qualitative findings on factors affecting the pricing decisions of wholesalers and retailers. We draw on an analytical framework based on the structure-conduct-performance (SCP) paradigm to suggest possible factors influencing antimalarial prices. Originally developed in the field of industrial organization (Bain 1956; Scherer and Ross 1990), the SCP paradigm has since been adapted for use in the health care sector to analyse hospital markets (Bennett 1996; Nakamba *et al*. 2002; Gaynor 2007) and retail markets for public health products (Conteh and Hanson 2003; Goodman 2004; Patouillard 2012). In our analytical framework (Figure 1) market performance outcomes are expressed in public health terms as the price, availability and quality of malaria treatment (Goodman 2004; Patouillard 2012). These outcomes are determined by a range of factors related to provider conduct, which in turn, both influence and are influenced by factors related to market structure and consumer demand. Consideration must also be given to contextual factors operating at national level, which could help to understand differences observed across countries. To illustrate, high consumer prices may result from limited price competition which may reflect more concentrated markets that arise due to high entry barriers, such as excessive business registration fees. Also, the way providers respond to pricing regulation may be influenced by the state’s ability to enforce these regulations, which could affect mark-ups and prices.

## Materials and methods

### Country contexts

*Plasmodium falciparum *is the dominant malaria species in all of the African study countries, and over 90% of the population in these countries live in areas of high transmission. In Cambodia, 44% of the population lives in high transmission areas and infections with *Plasmodium vivax *(*P. vivax*) account for over a third of malaria cases (Global Malaria Programme 2012). (Table 1).

**Table 1.** Key characteristics of malaria epidemiology, treatment policy and pharmaceutical licensing by country

	Country					
	Benin	Cambodia	DRC	Nigeria	Uganda	Zambia
Predominant malaria parasite species^a^	*P. falciparum* (100%)	*P. falciparum* (63%), *P. vivax* (37%)	*P. falciparum* (100%)	*P. falciparum* (100%)	*P. falciparum* (100%)	*P. falciparum* (100%)
% of population living in high transmission areas (≥1 case per 1000 population)^a^	100	44	97	100	90	100
Recommended first-line treatment for uncomplicated malaria (2010)^a^	AL	*P. falciparum:* ASMQ, DHA-PP-PQ; *P. vivax:* CQ, DHA-PP^b^	ASAQ	AL, ASAQ	AL	AL
ACT officially provided free of charge in public sector	No	Yes	Yes	Yes	Yes	Yes
Licences issued for pharmaceutical wholesaling	Yes: importer+wholesaler	Yes: importer, wholesaler+retailer	Yes: three types of wholesaler	Yes: importer, two types of wholesaler	Yes: wholesaler	Yes: importer, wholesaler
Licences issued for pharmaceutical retailing	Yes: retail pharmacy	Yes: wholesale+ retail pharmacy, depot A and B	Yes: retail pharmacy, hospital pharmacy	Yes: retail pharmacy	Yes: retail pharmacy	Yes: retail pharmacy
Licences issued for retailing of only OTC medicines	Yes: rural outpost pharmacy	No	No	Yes: PPMV	Yes: drug shop	Yes: drug store

*Notes: *P, *Plasmodium*; ACT, artemisinin combination therapy; AL, artemether-lumefantrine; ASAQ, artesunate–amodiaquine; ASMQ, artesunate–mefloquine; CQ, chloroquine; DHA-PP-PQ, dihydroartemisinin-piperaquine–primaquine; DHA-PP, dihydroartemisinin–piperaquine; OTC, over-the-counter.

^a^Source: Global Malaria Programme (2012).

^b^As part of the programme to contain the spread of artemisinin resistance, Cambodia’s treatment guidelines until early-2011 recommended the use of DHA-PP in the highest risk areas (combined with PQ where safe use has been demonstrated) and ASMQ everywhere else to treat *P. falciparum* malaria, and DHA-PP for the treatment of *P. vivax* malaria since 2011 (CQ was used previously). Since early-2011, Cambodia’s treatment guidelines have recommended the use of DHA-PP (combined with PQ where safe use has been demonstrated) for both *P. falciparum* and *P. vivax* malaria.

By the time of data collection in 2009–10, all study countries had adopted ACT as the first-line treatment for uncomplicated malaria. As part of the programme to contain the spread of artemisinin resistance in Cambodia, the national guidelines in effect during data collection recommended the use of dihydroartemisinin–piperaquine in areas where resistance had been detected and artesunate–mefloquine everywhere else to treat *P. falciparum *malaria, with chloroquine recommended for the treatment of *P. vivax *malaria (Dondorp *et al*. 2009). Although ACTs were being produced by domestic manufacturers in the DRC, Nigeria and Uganda, none of these products was prequalified by the World Health Organization at the time of data collection (Palafox *et al*. 2014). To further delay the development and spread of artemisinin resistance, all countries had banned or were phasing out the distribution of AMT in oral dosage forms.

In each study country, pharmaceutical regulatory authorities issue licences permitting businesses to wholesale and/or retail all registered pharmaceutical products. Apart from in Cambodia and the DRC, licences are also issued to operate smaller retail businesses authorizing them to sell a limited range of over-the-counter medicines. These retailers are typically known as drug shops, rural outpost pharmacies (*dépots de médicament*) in Benin or proprietary patent medicine vendors (PPMVs) in Nigeria. Many of these retail-only PPMVs are based in open-air markets in urban areas across Nigeria and often engage in unauthorized pharmaceutical wholesaling; and a similar situation has been observed among the unlicensed drug vendors operating open-air market stalls in Benin (Palafox *et al*. 2014).

The prices of pharmaceutical products are regulated in Benin and the DRC, although this power is not exercised by regulators in the DRC. In Benin, the basis for calculating private wholesaler prices is the manufacturer’s price before taxes, which excludes all taxes, transportation and insurance costs. Wholesalers are permitted to add a 36% mark-up on the manufacturer’s price, and retail pharmacies may add a 78% mark-up (or 31% mark-up on the wholesaler’s price). Rural outpost pharmacies may purchase stock from a single registered pharmacy at an 8% discount from the retail price (Tougher *et al*. 2009). Finally, although data collection was completed before the piloting of the Affordable Medicines Facility—malaria (AMFm) in 2011, sub-national private sector ACT subsidy programmes were ongoing in Nigeria (FMOH 2009) and Uganda (Talisuna *et al*. 2012), and there was a national ACT and RDT subsidy programme in Cambodia (Yeung *et al*. 2011).

### Quantitative methods

Retail outlets were sampled as part of the 2009–10 ACTwatch outlet survey (Littrell *et al*. 2011b; O'Connell *et al*. 2011), where 19 sub-districts/clusters in one to six strata were randomly sampled in each country using a probability-proportional-to-size approach. In each sub-district, a census of all public and private outlets that had the potential to dispense antimalarials was conducted. All outlets were screened for eligibility, with those stocking antimalarials or RDTs at the time of the survey or in the past 3 months invited to participate. In the African study countries, the main types of outlets sampled included public health facilities, private health facilities (both for- and not-for-profit), pharmacies, drug stores and other types of private outlets, such as grocery stores, kiosks, open-air market stalls in Benin and itinerant vendors in Nigeria and Benin. In Cambodia, health facilities and village malaria workers were the public sector outlet types sampled, and private sector outlet types included pharmacies/clinics (registered pharmacies, depots A and depots B; unregistered clinical pharmacies, cabinets and private clinics), unregistered drug stores, grocery stores in urban and rural areas, village shops in rural areas and mobile providers. These main samples were supplemented by over-sampling of both public facilities and retail pharmacies (including drug stores in the DRC, but only public facilities in Cambodia) which were relatively rare, in order to estimate antimalarial prices across retailer types. As this article concerns private sector prices, we present data from private for-profit outlets only. A total of 26 802 private outlets across six countries were censused to participate, of which 1650 could not be screened because they were closed down permanently, closed temporarily, an eligible provider was not available for interview, providers refused to participate or for other reasons. As a result, 25 152 outlets were screened for eligibility. Of these, 5788 met the eligibility criteria and were interviewed, during which 41 029 retail antimalarial products were inventoried (Table 2).

**Table 2.** Sample breakdown—number of retailers and wholesalers identified and interviewed, and antimalarial products audited

	Country					
	Benin	Cambodia	DRC	Nigeria	Uganda	Zambia
Dates of retail data collection for ACTwatch Outlet Survey	28 Apr–27 Jul 2009	9 Jun–8 Jul 2009	10 Aug–27 Oct 2009	4 Aug–16 Sep 2009	16 Mar–7 Apr 2009	14 Apr–3 Jul 2009
Dates of wholesale data collection ACTwatch Supply Chain Study	4–29 Jun 2009	21 Aug–1 Nov 2009	11 Jan–10 Mar 2010	18 Jul–8 Sep 2009	13 Feb–6 Apr 2009	28 Feb–6 May 2009
Number of outlets censused	1680	7287	3683	5727	4801	3624
Number of outlets screened	1488	7013	3604	5182	4652	3213
Number of outlets eligible	885	644	1303	1951	720	299
Number of outlets not interviewed	1	0	0	12	1	0
Number of quantitative interviews conducted at private outlets	884	644	1303	1939	719	299
Number of antimalarials inventoried at private outlets	4212	821	11 347	19 856	3955	838
Number of ACTwatch Outlet Survey clusters used to form terminal wholesaler sampling frame (over the total number of clusters)	19/19	20/38	32/76	20/76	38/38	38/38
Number of wholesalers identified through supplier mentions for the quantitative survey	228	141	179	213	170	57
Number of refusals	10	5	0	27	4	1
Number of duplicates	0	18	18	8	28	0
Number not eligible	1	9	1	5	1	9
Number not found	10	10	11	19	2	1
Number not interviewed for other reasons	3	4	10	14	6	2
Number of quantitative wholesaler interviews conducted	204^a^	95	139	140	129	44
Number of antimalarials inventoried at wholesale level	1529	230	1962	2600	1326	288
Number of qualitative in-depth interviews conducted with retailers	8	11	9	4	15	14
Number of qualitative in-depth interviews conducted with wholesalers^b^	19	22	14	26	18	18

^a^Results from Benin are weighted to adjust for any over- or under-sampling that may have occurred due to the high number of wholesalers operating within traditional markets.

^b^Many businesses identified as wholesale suppliers also operated as retailers (e.g. were retail pharmacies, drug stores or PPMVs selling medicines directly to consumers, particularly in Nigeria).

Wholesalers were sampled using an innovative bottom-up approach. We created the sampling frame for the first level of wholesalers, called ‘terminal wholesalers’ (i.e. wholesalers supplying outlets) using contact information for the top two antimalarial suppliers reported by private outlets, and any public facilities identifying private sector suppliers, as part of the ACTwatch outlet survey. In some smaller countries, all supplier information collected from the outlet survey sample was used to create the terminal-level sampling frame, and in other countries information from only a sub-sample of outlets was used (Table 2). We attempted to interview all wholesalers identified in the sampling frame. This process was repeated with all terminal wholesalers interviewed to identify businesses operating one level higher in the distribution chain (i.e. ‘intermediate-1 wholesalers’), and yet again (i.e. to identify ‘intermediate-2 wholesalers’, etc.) until only importers or manufacturers were identified as supply sources. At this point, it was deemed that the top of the distribution chain had been reached. Through this combination of censusing outlets and tracing their supply sources up through the distribution chain, we were able to identify all types of antimalarial retailer and wholesaler, including those that might otherwise be excluded because they do not possess the appropriate licence from regulatory authorities (e.g. unlicensed businesses, licensed retailers that wholesale). Using this method, 988 antimalarial wholesale sources operating at various distribution chain levels were identified. Of these, 26 were not eligible to participate because they did not have antimalarials or RDTs in stock at any point during the 3-month period prior to the survey, 10 were eligible but refused to participate, 125 were later found to be duplicate mentions or could not be found and a further 39 were not interviewed for other reasons. Across the six study countries, we conducted a total of 751 quantitative wholesaler interviews and inventoried 7935 wholesale antimalarial products.

In each eligible business, trained interviewers sought to speak with the person most knowledgeable about their antimalarial trade to obtain consent and administered data collection tools that were piloted and adapted for each country setting. A structured questionnaire was used to collect data on business characteristics, operations and top two supply sources of antimalarials. Inventory sheets were used to record each antimalarial stocked, including brand, generic name, strength, package type and size, recall of volumes sold over the week before the survey, recall of last purchase value and selling and purchase prices. Wholesaler data collection in each country was timed to follow shortly after outlet data collection and to coincide as much as possible with periods of peak malaria transmission (Table 2).

All data were double entered (Microsoft Access for outlet data and EpiData v.3.1 for wholesaler data) and analysed with Stata v.11 and v.12 (StataCorp 2009, 2011). Median retail prices are presented for adult equivalent treatment doses (AETDs), a standardized unit that allows for comparison of products with different treatment regimens, and converted to US dollars (USD) using the average annual exchange rate in 2009 for each country (O'Connell *et al*. 2011, 2013; Tougher *et al*. 2012). Retail percentage mark-ups were calculated as the difference between selling price and purchase price, divided by purchase price. Because it is common for wholesalers to vary their selling prices with the volumes being purchased, the median wholesale percentage mark-up presented is the mid-point mark-up calculated using the average of maximum and minimum selling prices charged for one unit relative to the price the wholesaler paid to purchase one unit. As such, the percentage mark-ups presented are gross mark-ups, reflecting both overhead costs and profit margins. Direct measurements of profit margins were not possible because collecting accurate data on provider costs proved too challenging.

Median point estimates are presented with their inter-quartile range (IQR). Retail-level estimates are weighted to account for the complex survey design, and wholesale-level estimates from Benin only are also weighted to account for any over- or under-sampling of wholesalers based in open-air markets (i.e. market stalls found to be wholesaling antimalarials) that may have occurred because of the challenges in identifying specific market stalls. Additional details on the rationale and approach to producing weighted estimates are provided in the Supplementary Text and elsewhere (Palafox *et al*. 2014). Missing price and mark-up data at both wholesale- and retail-level were not imputed as this was assumed to occur completely at random. The proportions of observations missing price and mark-up data are presented as footnotes in Tables 4 and 5.

### Qualitative methods

We conducted in-depth interviews with a subset of 117 antimalarial wholesalers and 61 retailers included in the quantitative surveys. These interviews were conducted at various levels of the distribution chain from manufacturers and importers down to retailers, and across various settings (i.e. urban, rural, accessible and remote) to capture a diverse range of experiences, practices and opinions (O'Cathain and Thomas 2006). A member of the research team conducted the interviews, with notes taken by a trained research assistant. A semi-structured topic guide was developed drawing on concepts from the analytical framework, and was used to ask participants to discuss their price setting practices and the factors that influence them.

Using a thematic analysis approach (Pope *et al*. 2006), all interview notes were read to identify the main themes or experiences. An initial coding structure was developed based on the analytical framework and existing literature, which was then applied to interview notes and revised as analysis proceeded by adding additional codes and sub-codes to capture as many nuances in the data as possible. Because one research team member coded all interviews for a given country, co-coding exercises were conducted at the beginning of the coding process to ensure consistency across countries, where pairs of researchers independently coded a minimum of five interview transcripts and then compared coding. Any discrepancies were discussed and agreed between coders (Pope *et al*. 2006). Data from related themes were grouped together and summarized by noting the frequency and range of terms, concepts, practices or experiences described by respondents. Differences across distribution chain levels and countries were noted. Coding and thematic analysis were conducted using NVivo 8 software. Information from these in-depth interviews was supplemented with a review of relevant documents on antimalarial regulation and policy.

### Ethical considerations

Ethical approval for this study was obtained from the London School of Hygiene & Tropical Medicine Ethics Committee (No. 5466, 18 February 2009) and from ethical review boards in each country. Free and informed consent was obtained verbally from all study participants.

## Results

In this section, we first present quantitative data on retail prices and percentage mark-ups at retail and wholesale level. Prices are disaggregated by antimalarial type and dosage form. Retail mark-ups are shown first by antimalarial type so that the main patterns of difference across countries can be seen; and then disaggregated by outlet type for ACT tablets only (data for other ACT dose forms are provided in Supplementary Table S1, and for AMTs and nATs are available at www.actwatch.info). Wholesale mark-ups are disaggregated by antimalarial type and dose form. This is followed by qualitative data on the price setting behaviour of wholesalers and retailers.

### Prices and mark-ups

#### Retail prices

Within each country, median retail prices per AETD tended to be lowest on nATs, followed by ACTs and then AMTs (Table 3). Products in tablet form tended to have lower prices per AETD than those in oral liquid and injectable form. However in the African study countries, retail prices for AMTs in tablet and oral liquid forms were often similar to or lower than those for ACTs.

**Table 3.** Median retail selling prices by country (private outlets only), antimalarial type and dosage form (USD)

Antimalarial type	Formulation^a^		Country					
			Benin	Cambodia	DRC	Nigeria	Uganda	Zambia
			*N* = 643	*N* = 405	*N* = 1264	*N* = 1678	*N* = 685	*N* = 259
**ACT**	All	Median	8.29	1.18	3.51	3.86	4.73	9.36
		IQR	5.84–13.28	0.94–1.88	1.46–4.86	2.57–5.46	2.37–7.45	5.62–13.10
		(*n*)	(1927)	(454)	(2597)	(4956)	(437)	(234)
	Tablet	Median	7.50	1.18	3.03	3.86	4.26	8.98
		IQR	5.51–9.32	0.94–1.88	1.46–4.25	2.57–5.01	2.27–6.62	5.62–10.29
		(*n*)	(1501)	(454)	(1976)	(4001)	(380)	(186)
	Oral liquid	Median	20.00	—	11.65	10.29	17.03	17.47
		IQR	18.06–22.13	—	8.74–14.24	7.71–12.00	12.62–18.93	13.9–24.96
		(*n*)	(390)	(0)	(572)	(715)	(48)	(48)
**AMT**	All	Median	24.28	4.52	5.83	3.54	11.36	33.69
		IQR	14.32–49.72	3.01–12.71	3.03–9.71	2.57–9.64	7.57–15.14	6.74–34.94
		(*n*)	(288)	(187)	(2526)	(2477)	(393)	(84)
	Tablet	Median	9.45	3.62	3.03	3.09	9.08	6.29
		IQR	7.74–16.57	2.64–4.52	2.19–3.88	2.57–3.60	7.57–11.36	5.33–6.29
		(*n*)	(57)	(129)	(955)	(1438)	(229)	(16)
	Oral liquid	Median	21.21	—	8.16	13.37	15.14	5.99
		IQR	20.58–23.21	—	6.99–13.84	9.26–17.36	12.11–15.14	5.99–8.98
		(*n*)	(20)	(0)	(1071)	(691)	(32)	(23)
	Injectable	Median	49.72	22.60	12.75	11.57	17.03	33.69
		IQR	26.48–49.72	15.07–26.36	8.74–15.86	9.26–15.43	11.36–22.71	22.71–44.92
		(*n*)	(131)	(57)	(490)	(340)	(127)	(45)
**nAT**	All	Median	0.62	0.46	2.75	0.80	1.42	0.47
		IQR	0.31–3.23	0.23–7.41	0.49–6.12	0.45–1.25	0.46–3.97	0.34–1.36
		(*n*)	(1897)	(88)	(6095)	(10630)	(3015)	(514)
	Tablet	Median	0.39	0.41	0.73	0.51	0.48	0.45
		IQR	0.26–2.12	0.23–0.46	0.36–3.06	0.32–0.77	0.34–1.99	0.28–0.66
		(*n*)	(1385)	(68)	(3530)	(5789)	(1694)	(418)
	Oral liquid	Median	1.86	—	7.65	1.16	3.55	4.49
		IQR	1.24–3.11	—	2.91–12.24	0.90–1.61	1.18–6.11	3.18–7.86
		(*n*)	(271)	(0)	(2150)	(4284)	(1043)	(76)
	Injectable	Median	15.23	9.89	6.37	0.60	9.94	23.58
		IQR	8.70–22.03	7.41–14.83	6.12–7.65	0.40–0.90	6.96–14.90	9.83–31.45
		(*n*)	(241)	(20)	(398)	(557)	(278)	(20)

*Notes*: *N*, total number of retail outlets from which pricing data was obtained; *n*, total number of individual antimalarial products audited contributing to the calculation of the weighted median. Retail price data were missing from 2.4% of audited products in Benin, 10.5% in Cambodia, 1.1% in the DRC, 9.0% in Nigeria, 2.8% in Uganda and 0.7% in Zambia.

^a^The values for median price reported for ‘all’ formulations include all dosage forms (tablets, suppositories, oral liquids, injectables and granules); however, because so few wholesalers and retailers stocked granules or suppositories, and so few of these product types were observed during the audit, results are not presented separately for these categories in this table.

**Table 4.** Median percentage mark-ups on ACT tablets, retail level (%)

Country		Retailer Categories			
		Pharmacies	Private health facilities^a^	Drug stores	Other private outlets^a^
Benin		*N* = 28	*N* = 62	*N* = 0	*N* = 350
	Median	30.9	33.3	—	16.7
	IQR	30.8–31.0	22.5–100.0	—	−20.0–33.3^b^
	(*n*)	(231)	(18)	(0)	(9)
DRC		*N* = 30	*N* = 112	*N* = 945	*N* = 16
	Median	33.3	30.6	38.9	25.0
	IQR	20.0–66.7	10.0–50.0	25.0–66.7	22.8–100.0
	(*n*)	(148)	(51)	(1415)	(7)
Nigeria		*N* = 274	*N* = 156	*N* = 906	*N* = 96
	Median	25.0	41.7	23.8	29.0
	IQR	16.7–37.5	8.3–66.7	14.6–40.0	16.7–42.9
	(*n*)	(1669)	(159)	(690)	(36)
Uganda		*N* = 89	*N* = 173	*N* = 349	*N* = 9
	Median	42.9	42.9	40.0	—
	IQR	23.1–60.0	11.1–76.5	25.0–100.0	—
	(*n*)	(202)	(56)	(47)	(0)
Zambia		*N* = 39	*N* = 27	*N* = 92	*N* = 32
	Median	42.9	71.4	233.3	
	IQR	36.4–66.7	33.3–89.7	84.6–248.8	N/S
	(*n*)	(95)	(16)	(12)	


Country		Retailer categories				
		Pharmacies and clinics	Drug stores	Mobile providers	Grocery stores	Village shops
Cambodia		*N* = 77	*N* = 75	*N* = 101	*N* = 57	*N* = 72
	Median	40.0	50.0	50.0	40.0	28.6
	IQR	20.0–80.0	25.0–106.9	25.0–66.7	20.0–60.0	14.3–55.6
	(*n*)	(119)	(85)	(104)	(51)	(59)


*Notes*: *N*, total number of retail outlets from which pricing data was obtained; *n*, total number of individual antimalarial products audited contributing to the calculation of the weighted median; N/S, result not shown due to insufficient observations (*n* < 5) to obtain a reliable estimate. Because of missing data, mark-ups could not be calculated for 68.3% of audited products in Benin, 17.2% in Cambodia, 17.3% in the DRC, 33.9% in Nigeria, 16.6% in Uganda and 30.9% in Zambia.

^a^Private health facilities include both for-profit and not-for-profit facilities; Other private outlets include supermarkets, kiosks, itinerant medicine sellers (hawkers) and outlet types that do not fit into any of the mentioned outlet categories.

^b^A negative mark-up estimate for the lower IQR bound indicates that ACTs were being sold at a loss.

**Table 5.** Median percentage price mark-ups at wholesale level, by country, antimalarial type and dosage form

Antimalarial type	Formulation^a^		Country					
			Benin	Cambodia	DRC	Nigeria	Uganda	Zambia
			*N* = 199	*N* = 78	*N* = 135	*N* = 136	*N* = 127	*N* = 40
ACT	All	Median	31.0	41.2	11.1	17.6	14.3	26.7
		IQR	25.0–36.0	25.0–66.7	5.8–22.7	10.7–33.3	7.1–25.0	20.0–39.5
		(*n*)	(378)	(129)	(685)	(753)	(277)	(58)
	Tablet	Median	31.0	41.2	11.1	17.6	14.3	24.2
		IQR	20.0–36.0	25.0–66.7	6.4–25.0	11.1–35.2	7.1–25.0	18.8–34.6
		(*n*)	(308)	(129)	(472)	(596)	(232)	(41)
	Oral liquid	Median	33.0	—	11.1	16.7	14.8	29.7
		IQR	30.9–36.0	—	5.2–21.1	9.8–26.7	6.7–22.2	25.0–42.9
		(*n*)	(55)	(0)	(202)	(111)	(38)	(17)
AMT	All	Median	36.0	16.7	11.1	20.0	15.0	26.1
		IQR	31.2–36.0	7.3–29.2	6.5–20.0	10.0–37.5	7.5–28.6	18.0–33.8
		(*n*)	(81)	(28)	(273)	(358)	(293)	(32)
	Tablet	Median	36.0	16.7	11.2	22.1	15.4	25.5
		IQR	30.9–36.0	6.2–25.0	9.3–18.9	11.1–40.7	7.7–29.8	21.6–42.5
		(*n*)	(16)	(15)	(44)	(181)	(162)	(9)
	Oral liquid	Median	36.0	—	11.1	14.6	15.4	31.0
		IQR	36.0–36.0	—	6.6–17.2	8.8–31.6	8.3–29.2	20.6–35.0
		(*n*)	(6)	(0)	(63)	(106)	(27)	(8)
	Injectable	Median	36.0	20.0	11.1	21.4	11.8	25.0
		IQR	31.0–36.0	11.8–42.9	5.5–22.0	11.1–35.3	6.3–22.7	11.1–33.0
		(*n*)	(39)	(13)	(161)	(69)	(103)	(15)
nAT	All	Median	31.0	29.3	12.4	25.0	17.6	24.7
		IQR	16.7–36.0	14.0–60.0	6.8–25.0	11.4–47.7	8.6–35.1	13.0–50.0
		(*n*)	(733)	(29)	(634)	(915)	(623)	(53)
	Tablet	Median	29.2	42.3	12.4	25.0	15.4	24.7
		IQR	15.4–36.0	20.0–100.0	6.4–25.0	11.1–42.9	6.7–35.0	14.6–50.0
		(*n*)	(530)	(23)	(354)	(534)	(300)	(43)
	Oral liquid	Median	33.0	—	12.2	29.2	20.0	25.0
		IQR	16.6–36.0	—	8.5–25.0	12.5–50.0	9.4–38.9	13.0–50.0
		(*n*)	(117)	(0)	(210)	(306)	(254)	(9)
	Injectable	Median	36.0	15.6	13.3	25.7	20.0	
		IQR	22.7–36.0	5.9–25.0	5.1–25.0	15.4–54.3	11.1–29.2	N/S
		(*n*)	(84)	(6)	(61)	(71)	(66)	

*Notes*: *N*, total number of wholesalers from which pricing data was obtained; *n*, total number of individual antimalarial products audited contributing to the calculation of the median; N/S, result not shown due to insufficient observations (*n* < 5) to obtain a reliable estimate. Because of missing data, mark-ups could not be calculated for 20.0% of audited products in Benin, 26.3% in Cambodia, 19.0% in the DRC, 22.0% in Nigeria, 10.0% in Uganda and 49.7% in Zambia.

^a^The values for median mark-up reported for ‘all’ formulations include all dosage forms (tablets, suppositories, oral liquids, injectables and granules); however, because so few wholesalers and retailers stocked granules or suppositories, and so few of these product types were observed during the audit, results are not presented separately for these categories in this table.

Comparing across countries, the retail price per AETD for ACT tablets was lowest in Cambodia (USD 1.18); followed by the DRC, Nigeria and Uganda (USD 4.03 to USD 4.26); and highest in Benin (USD 7.50) and Zambia (USD 8.98). In contrast, the retail price per AETD for nAT tablets was much lower and more consistent across countries, ranging from USD 0.39 in Benin to USD 0.73 in the DRC.

#### Retail mark-ups

**Figure 2.**
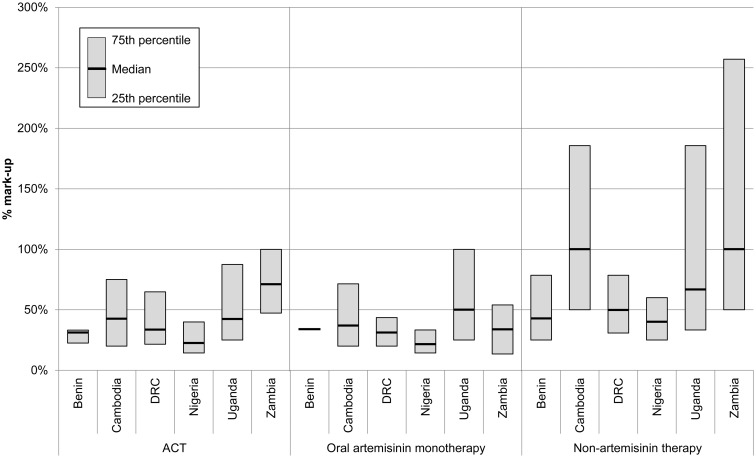
Median percentage price mark-ups and IQR at retail level by country and antimalarial type

Retailers in all countries tended to apply the highest percentage mark-ups on nATs, ranging from 40% in Nigeria to 100% in Cambodia and Zambia (Figure 2). In all countries except Zambia, mark-ups on ACTs and oral AMTs were similar in magnitude and tended to be lower than those applied to nATs. Median mark-ups on ACTs ranged from 22% in Nigeria to 71% in Zambia; and on oral AMTs from 22% in Nigeria to 50% in Uganda.

Comparing across countries, retailers in Nigeria tended to apply the lowest mark-ups on all antimalarial types. The highest mark-ups on ACTs and nATs were applied by retailers in Zambia, and Ugandan retailers applied the highest mark-ups on oral AMTs. However, in some countries, these national-level summary statistics mask notable variation across retailer types (Table 4 and Supplementary Table S1). For example, the median mark-up on ACT tablets in Zambia ranged from 43% in pharmacies to 233% in drug stores. In Benin pharmacies consistently applied mark-ups of 31% (IQR 31–31%) across all antimalarial types (Supplementary Table S1 for ACTs, data for AMTs and nATs not shown), whereas the mark-ups applied by other types of retailers varied considerably. For example, at private health facilities in Benin the IQR of mark-ups on nATs ranged from 33% to 100% (*n* = 157), and ranged from 25% to 66% (*n* = 625) at ‘other private outlets’.

#### Wholesale mark-ups

Median wholesale percentage mark-ups on antimalarials varied by country (Table 5). They were lowest in the DRC (ranging from 11% to 13%) and Uganda (14% to 20%), followed by Nigeria (15% to 30%) and Zambia (25% to 31%), and highest in Benin (31% to 36%). Mark-ups at wholesale level were generally lower than those at retail level.

Unlike at retail level, median wholesale mark-ups were fairly consistent across antimalarial categories in the African study countries. In Cambodia, wholesale mark-ups were more variable, and tended to be lower for AMTs (17%) and higher for ACTs (41%). In all countries, wholesale mark-ups tended to be consistent across dosage forms.

More detailed analysis in Benin revealed that wholesalers operating outside of open-air markets, such as registered wholesalers, applied mark-ups that were largely consistent with price setting regulation (i.e. 36% on purchase prices), whereas market-based wholesalers (i.e. market stalls wholesaling antimalarials) applied mark-ups that were considerably lower, ranging from a median of 13% (IQR 5-25) on ACT, to 19% (IQR 11-31) on nAT.

### Exploring price-setting behaviour

#### Operating expenses

During qualitative interviews, respondents listed a wide range of factors that they considered when setting prices or that they believed influenced antimalarial prices. In all countries, respondents commonly mentioned supplier prices and the cost of transporting goods as important factors. These, in turn, were said to be affected by USD exchange rate volatility, which was of particular concern among respondents from the DRC, Nigeria, Uganda and Zambia. Other operating expenses were also considered when setting prices. These included the costs of renting and maintaining premises, and in the African study countries, salaries. Wholesalers in Benin and Nigeria also counted telephone charges as significant recurrent expenses. Outlays for maintaining pharmaceutical licenses and professional registration were mentioned by several respondents in all countries except Zambia; however, unlicensed businesses, such as market stalls and small drug shops engaged in unauthorized wholesaling, largely avoided these expenses. Because many antimalarial vendors in Cambodia operated as kiosks at the front of the owners’ homes, these businesses did not incur expenses for rent, salaries, etc.

#### Price competition and sensitivity

Competition was another important consideration for many respondents when setting antimalarial prices. In most countries, competition was perceived to be intense, with both wholesalers and retailers often saying that they must adhere to prevailing market prices. To illustrate, respondents in several countries said they regularly surveyed competitors’ prices by visiting other businesses and by asking customers what competitors were charging for similar products. In Cambodia and the DRC, respondents also described consumers as very price sensitive. Although this was a more immediate concern for retailers when setting prices, some wholesalers also considered consumer price sensitivity. As an important client retention strategy, these wholesalers said they were prepared to lower their mark-ups so that the retailers they supplied could still make decent profits.

#### Product availability and characteristics

Product scarcity was another factor affecting price mentioned in several countries, which was reported to fuel dramatic price increases. For example, both retailers and wholesalers in Uganda described the difficulties of finding suppliers of chloroquine as a result of regulatory efforts to reduce domestic production and importation, and shift demand towards ACT. Several wholesalers commented on how the scarcity of some nATs, combined with persistent high demand, caused their prices to escalate. Many respondents expressed concern over this trend because, in the absence of affordable alternatives, rising prices reduced access to treatment more broadly.

Other factors affecting price were related to product characteristics. For example, domestically produced antimalarials were often said to attract lower mark-ups than imported products, whereas higher mark-ups could be applied to popular products compared with those in less demand. A number of respondents also described how approaching product expiration dates led many businesses to reduce mark-ups in order to induce sales and avoid losses.

#### Discounting and price discrimination

Price discounts were a regular tool used by wholesalers and retailers in all countries. Wholesalers commonly used discounts based on order volume or value. In most cases, customers had to reach a certain threshold before qualifying for discounts, and in some instances the size of the discount, usually a small percentage of the total order value, was linked to the order size. Both wholesalers and retailers also offered discounts to certain types of customers. Some wholesalers in Benin and Nigeria described giving discounts to customers paying in cash, rather than to those using supplier credit facilities. Some wholesalers in Benin, the DRC, Nigeria and Uganda also offered discounts to regular or long-term customers. Although not strictly a discount, retailers in most countries reported considering a consumer’s ability to pay when deciding on a price. Several retail respondents even described giving antimalarials free of charge when they felt a patient could not afford to purchase treatment.

#### Price regulation and other constraints

Although legislation for pharmaceutical price regulation existed in Benin and the DRC, respondents in the DRC said that such regulation was only nominally present and had no effect on their pricing decisions. Some of these respondents admitted that they were not aware of its existence. In Benin, however, respondents from businesses operating outside of open-air markets (e.g. pharmacies) described applying mark-ups that adhered to the pricing regulations, whereas respondents operating open-air market stalls did not. In most countries respondents mentioned other types of pricing constraints, including compulsory pricing imposed by suppliers (i.e. vertical restraints) and the addition of recommended retail prices (RRP) on product packaging. Although respondents in most countries mentioned these practices, many agreed that these pricing constraints were not adhered to or were sometimes viewed more as pricing guidelines rather than as rules. Even in Cambodia, where the brand of first-line treatment, Malarine, had been socially marketed for a number of years with the RRP printed on the package, very few retailers reported setting their price at the recommended level, arguing that the RRP was too low and did not provide a sufficient margin on top of the wholesale purchase price.

## Discussion

There are several important limitations to consider when interpreting the findings from this study. First, data collection pre-dated implementation of the AMFm, which intervened in antimalarial markets through a high-level subsidy on ACTs distributed through both public and private sector outlets, and led to large reductions in ACT prices in the private for-profit sector in most settings (Tougher *et al*. 2012). However, the AMFm was only piloted in seven countries over the period 2010–12, and other large scale ACT subsidy programmes remain few in number, meaning that most malaria-endemic countries do not have such programmes operating at national scale. Although smaller ACT price reductions have also been documented in the absence of ACT subsidies since 2009 (Tougher *et al*. 2012), it remains likely this study’s findings on prices and mark-ups continue to represent the broad picture in antimalarial markets in many settings. Furthermore, one would expect many of the pricing determinants identified through our qualitative work to be relevant in settings both with and without ACT subsidies.

The potential bias on mark-up estimates arising from missing data is another concern. Over 30% of mark-up data were missing at retail level in Benin, Nigeria and Zambia, and at wholesale level in Zambia (see footnotes to Tables 4 and 5), likely reflecting problems of recall and the perceived commercial or legal sensitivity of these data. There is some evidence that the pattern of missing data varies by retailer type and dosage form, which could bias the retail mark-up estimates aggregated across these variables shown in Figure 2. Our investigation of the pattern of missing data suggests that in most cases, biases are likely to have cancelled each other out. However, the median retail mark-ups for nATs in Figure 2 may be underestimated. This source of bias is likely to have had a much smaller impact on estimates when disaggregated by dosage form and outlet type (retail level only) as in Tables 4 and 5. We elected not to impute missing mark-up data because this potential bias does not change the overall pattern or interpretation of our findings; furthermore, small cell sizes can contribute to unreliable or inconclusive imputations, leading to further problems of interpretation.

Social desirability bias may also have led to underestimates of prices and mark-ups, and under-reporting of questionable businesses practices during qualitative interviews. We designed tools and methods to minimize the impact of such bias. For example, during introductions interviewers assured participants that we were not connected with regulatory authorities and that participants would not be identified; we also structured qualitative interviews to ask more sensitive questions towards the end when a rapport with the respondent was more likely to have been established. Errors in price measurement may have occurred because of poor recall or in cases where unit prices had to be estimated for bulk orders. Finally, missing supplier information from retail outlets may have also biased wholesaler sampling frames towards registered suppliers; however, our ‘bottom-up’ sampling approach did identify considerable numbers of unregistered wholesalers that were included in each of our country samples.

Despite these limitations, this study has produced rigorous estimates of antimalarial prices and mark-ups, and has bettered our understanding of their determinants. In the African study countries retail prices for ACT and oral AMT were similar, but prices were many times higher than for nATs, whereas in Cambodia the price difference between ACTs and nATs was much less pronounced. Percentage mark-ups at retail level varied by outlet type, but were similar for ACTs and oral AMTs, and comparatively lower than those applied to nATs. In contrast, wholesale mark-ups were more consistent across antimalarial type and tended to be lower than those at retail level. The latter is unsurprising as the much larger sales volumes handled by wholesalers lead to lower unit costs. Although few studies of antimalarial mark-ups have been previously conducted, these patterns are broadly consistent with findings reported in a 2010 literature review (Patouillard *et al*. 2010). However compared with our findings, the review reported a much wider range of mark-ups both at wholesale level (2–99%) and retail level (up to 566% in pharmacies, 669% in drug shops and 233% in general shops). These differences are likely related to varying methods for estimating mark-ups and to differences in study scope and size.

One notable observation from this study is that both wholesale and retail mark-ups were comparable to those permitted in countries at similar levels of development that regulate pharmaceutical mark-ups (Ball 2011). This suggests that mark-ups are not excessive, and that price gouging or setting prices above the market price is not a widespread practice. This is supported by our qualitative finding that antimalarial trading was competitive among retailers and wholesalers, and reflected in their willingness to reduce mark-ups in order to offer discounts as a customer retention strategy.

Our findings suggest that in addition to competition, other elements of market structure, provider conduct, consumer demand and context were also important influences on pricing behaviour. The observed variation in mark-ups, particularly across different retailer types, may be partly related to the diversity of businesses dispensing antimalarials and the operating costs they incur. This was most plainly demonstrated in Benin where wholesalers and retailers operating outside of open-air markets applied percentage mark-ups that were largely consistent with price regulations (i.e. 31–36% on purchase prices), whereas open-air market traders applied considerably lower mark-ups. Benin’s pricing regime is designed to allow margins that adequately cover operating costs while generating modest profits, but because open-air market traders operate outside of the regulatory framework (Palafox *et al*. 2014), they likely avoid many of the overhead expenses incurred by their licensed counterparts, such as pharmacist salaries and licensing fees. They are also likely to have comparatively lower costs associated with rent and utilities.

Wholesalers tended to apply similar percentage mark-ups on all antimalarials. In contrast, retailers followed a commonly mentioned pricing strategy by applying higher mark-ups on nATs, the most popular type of antimalarial dispensed in the African study countries (Littrell *et al*. 2011b). Although nATs tended to attract the highest mark-ups in percentage terms, their considerably lower prices meant that the absolute revenue gained from the sale of each nAT was still small in currency terms. This suggests that, unlike wholesalers, retailers differentiate between antimalarials, viewing ACTs and AMTs more as luxury products and marketed according to a low-volume high-margin sales model, whereas nATs are treated more as commodity products being sold on a high-volume low-margin basis (McCabe *et al*. 2011). This market segmentation is not unlike that observed between generic and innovator pharmaceutical products in both developed and developing country markets (Scherer 1993; Frank and Salkever 1997; Nunn *et al*. 2009). Therefore, shifting ACTs from one sales model to the other could be key to increasing their access through the private for-profit sector.

One of the largest barriers impeding this shift is the persistently high price of ACT relative to other types of antimalarials. Our findings suggest that reducing commodity costs to retailers would be the most immediate way to improve ACT affordability. This was the motivation behind several subsidy interventions, most notably the AMFm. One concern for the AMFm was whether the value of the subsidy would be consumed by various middlemen operating at subsequent levels of the distribution chain (Laxminarayan and Gelband 2009). However, consumer ACT prices did fall substantially in most AMFm pilot countries (Tougher *et al*. 2012), which implies that—consistent with our findings—wholesalers and retailers were not engaging in excessive price gouging, in contexts where adequate supplies existed and markets were relatively competitive.

Given the cost and complexity of implementing large-scale subsidy programmes, other approaches may also be considered for reducing wholesale ACT prices. These include pooled purchasing mechanisms that would allow groups of retailers to benefit from supplier volume discounts, and interventions that increase wholesaler coverage and reduce the number of supply chain steps between manufacturer and retailer (Palafox *et al*. 2014). More modest price reductions might also be achieved by helping businesses lower operational expenses, particularly those related to transport. Finally, while price setting regulation can be effective in some market segments, its impact is likely to be limited to businesses normally subjected to high levels of oversight. In settings where unlicensed wholesalers and retailers play a large role and/or where regulatory capacity is low, well publicized RRPs could present a more effective means of ensuring price restraint in some circumstances.

## Conclusion

In the six study countries, retail-level percentage mark-ups for ACT and oral AMT were similar, and lower than those applied to nATs. Wholesale-level mark-ups were more consistent across antimalarial type and lower than those observed at retail level. When determining antimalarial prices and mark-ups, wholesalers and retailers consider a range of factors related to operating expenses, competition, product availability, product characteristics and to a lesser degree price regulation and other pricing constraints. In general, mark-ups both at retail and wholesale level were not excessive, suggesting that reducing commodity costs to retailers has the greatest potential to improve ACT affordability in the private for-profit sector. This could most directly be achieved through ACT subsidies, with pooled purchasing or interventions to improve wholesaler coverage also likely to be of value.

## Supplementary data

Supplementary data are available at *HEAPOL* online

Supplementary Data
