# Supplementary material for: Prices and mark-ups on antimalarials: evidence from nationally representative studies in six malaria-endemic countries
Source: Health Policy Plan. 2015 May 5;31(2):148–60. doi: 10.1093/heapol/czv031 (PMC4748126; doi:10.1093/heapol/czv031)
Supplement: Supplementary Data [file supp_31_2_148__index.html]

Prices and mark-ups on antimalarials: evidence from nationally representative studies in six malaria-endemic countries — Supplementary Data 

# Prices and mark-ups on antimalarials: evidence from nationally representative studies in six malaria-endemic countries

## Supplementary Data

files

**Files in this Data Supplement:**

- Supplementary Data - doc file
- Supplementary Data - doc file
