# Supplementary material for: Prices and mark-ups on antimalarials: evidence from nationally representative studies in six malaria-endemic countries
Source: Health Policy Plan. 2015 May 5;31(2):148–60. doi: 10.1093/heapol/czv031 (PMC4748126; doi:10.1093/heapol/czv031)
Supplement: Supplementary Data [file supp_czv031_Supplementary_Text_-_REVISED_20141210.doc]

*Supplementary Text: Rationale and approach to calculating weighted price and mark-up estimates*

Retail-level medians and IQRs were weighted using the *aweight* option in Stata to account for the stratified and clustered sampling strategy, which involved a census in sub‐districts of varying size selected using PPS and to adjust for over-sampling of pharmacies. Weights were based on the inverse of the probability of selection where the distribution of outlets was assumed to be proportional to population size.

Wholesale-level findings from Benin were also weighted to account for any over‐ or under-sampling of wholesalers based in open-air markets that may have occurred. While such wholesalers were also commonly observed in Nigeria, identifying wholesalers to interview in Benin using the bottom-up approach proved challenging as many supplier mentions included only a market name as the source of antimalarials, rather than naming a specific business. Ascertaining how many wholesalers to interview in each market also posed difficulties as registers of these businesses which might have been used as sampling frames do not exist. As such, pseudo-probability weights for each identified market were calculated using the total number of supplier mentions collected to approximate the distribution of wholesalers based in open-air markets, and applied in a similar way to obtain medians and IQRs. See Palafox et al. 2014 for further details.
