# Supplementary material for: Prices and mark-ups on antimalarials: evidence from nationally representative studies in six malaria-endemic countries
Source: Health Policy Plan. 2015 May 5;31(2):148–60. doi: 10.1093/heapol/czv031 (PMC4748126; doi:10.1093/heapol/czv031)
Supplement: Supplementary Data [file supp_czv031_Supplementary_Table_A1-REVISED_20141210.doc]

*Supplementary Table A1: Median percent mark-ups on ACTs by dosage form, retail level (%)*

| **COUNTRY**  Formulation | | | | | **RETAILER CATEGORIES** | | | |
| --- | --- | --- | --- | --- | --- | --- | --- | --- |
|  |  |  |  |  | **PHARMACIES** | **PRIVATE HEALTH FACILITIES**^4^ | **DRUG STORES** | **OTHER PRIVATE OUTLETS^4^** |
| **BENIN** | | | | | **N=28** | **N=62** | **N=0** | **N=350** |
|  | | All | | **Median** | **30.9** | **33.3** | **-** | **16.7** |
|  |  |  |  | IQR | 30.9-31.0 | 22.5-83.3 | - | -20.0-33.3 |
|  |  |  |  | (n) | (307) | (23) | (0) | (10) |
|  |  | Tablet | | **Median** | **30.9** | **33.3** | **-** | **16.7** |
|  |  |  |  | IQR | 30.8-31.0 | 22.5-100.0 | - | -20.0-33.3 |
|  |  |  |  | (n) | (231) | (18) | (0) | (9) |
|  |  | Oral liquid | | **Median** | **30.9** |  | **-** |  |
|  |  |  |  | IQR | 30.9-31.0 | N/S | - | N/S |
|  |  |  |  | (n) | (67) |  | (0) |  |
| **DRC** | | | | | **N=30** | **N=112** | **N=945** | **N=16** |
|  | | All | | **Median** | **33.3** | **30.6** | **35.1** | **25.0** |
|  |  |  |  | IQR | 20.0-57.7 | 11.1-50.0 | 22.4-66.7 | 22.8-100.0 |
|  |  |  |  | (n) | (203) | (64) | (1862) | (8) |
|  |  | Tablet | | **Median** | **33.3** | **30.6** | **38.9** | **25.0** |
|  |  |  |  | IQR | 20.0-66.7 | 10.0-50.0 | 25.0-66.7 | 22.8-100.0 |
|  |  |  |  | (n) | (148) | (51) | (1415) | (7) |
|  |  | Oral liquid | | **Median** | **23.5** | **31.6** | **29.4** |  |
|  |  |  |  | IQR | 13.2-33.3 | 13.9-39.0 | 17.6-36.8 | N/S |
|  |  |  |  | (n) | (47) | (13) | (416) |  |
|  |  | Granule | | **Median** | **29.6** | **-** | **33.3** | **-** |
|  |  |  |  | IQR | 20.0-29.6 | - | 23.8-42.9 | - |
|  |  |  |  | (n) | (8) | (0) | (31) | (0) |
| **NIGERIA** | | | | | **N=274** | **N=156** | **N=906** | **N=96** |
|  | All | | **Median** | | **25.0** | **41.7** | **22.2** | **29.0** |
|  |  |  | IQR | | 16.9-31.0 | 12.5-66.7 | 13.3-40.0 | 18.2-42.9 |
|  |  |  | (n) | | (2150) | (210) | (783) | (39) |
|  | Tablet | | **Median** | | **25.0** | **41.7** | **23.8** | **29.0** |
|  |  |  | IQR | | 16.7-37.5 | 8.3-66.7 | 14.6-40.0 | 16.7-42.9 |
|  |  |  | (n) | | (1669) | (159) | (690) | (36) |
|  | Oral liquid | | **Median** | | **16.9** | **25.0** | **15.4** |  |
|  |  |  | IQR | | 16.9-22.2 | 16.7-40.0 | 9.0-30.4 | N/S |
|  |  |  | (n) | | (333) | (31) | (71) |  |
|  | Granule | | **Median** | | **25.0** | **33.3** | **25.0** | **-** |
|  |  |  | IQR | | 25.0-25.0 | 25.0-50.0 | 19.0-40.0 | - |
|  |  |  | (n) | | (131) | (19) | (19) | (0) |
| **UGANDA** | | | | | **N=89** | **N=173** | **N=349** | **N=9** |
|  | All | | **Median** | | **42.9** | **43.6** | **33.3** | **-** |
|  |  |  | IQR | | 23.1-52.5 | 15.4-76.5 | 20.0-100.0 | - |
|  |  |  | (n) | | (240) | (61) | (48) | (0) |
|  | Tablet | | **Median** | | **42.9** | **42.9** | **40.0** | **-** |
|  |  |  | IQR | | 23.1-60.0 | 11.1-76.5 | 25.0-100.0 | - |
|  |  |  | (n) | | (202) | (56) | (47) | (0) |
|  | Oral liquid | | **Median** | | **50.0** |  |  | **-** |
|  |  |  | IQR | | 25.0-50.0 | N/S | N/S | - |
|  |  |  | (n) | | (30) |  |  | (0) |
| **ZAMBIA** | | | | | **N=39** | **N=27** | **N=92** | **N=32** |
|  | All | | **Median** | | **42.9** | **71.4** | **200.0** |  |
|  |  |  | IQR | | 37.0-66.7 | 47.4-89.7 | 84.6-248.8 | N/S |
|  |  |  | (n) | | (122) | (22) | (15) |  |
|  | Tablet | | **Median** | | **42.9** | **71.4** | **233.3** |  |
|  |  |  | IQR | | 36.4-66.7 | 33.3-89.7 | 84.6-248.8 | N/S |
|  |  |  | (n) | | (95) | (16) | (12) |  |
|  | Oral liquid | | **Median** | | **42.9** | **71.4** |  | **-** |
|  |  |  | IQR | | 42.1-59.1 | 47.4-94.4 | N/S | - |
|  |  |  | (n) | | (27) | (6) |  | (0) |

| **COUNTRY**  Formulation | | | **RETAILER CATEGORIES** | | | | |
| --- | --- | --- | --- | --- | --- | --- | --- |
|  |  |  | **PHARMACIES & CLINICS** | **DRUG STORES** | **MOBILE PROVIDERS** | **GROCERY STORES** | **VILLAGE SHOPS** |
| **CAMBODIA** | | | **N=77** | **N=75** | **N=101** | **N=57** | **N=72** |
|  | All | **Median** | **40.0** | **50.0** | **50.0** | **40.0** | **28.6** |
|  |  | IQR | 20.0-80.0 | 25.0-106.9 | 25.0-66.7 | 20.0-60.0 | 14.3-55.6 |
|  |  | (n) | (119) | (85) | (104) | (51) | (59) |
|  | Tablet | **Median** | **40.0** | **50.0** | **50.0** | **40.0** | **28.6** |
|  |  | IQR | 20.0-80.0 | 25.0-106.9 | 25.0-66.7 | 20.0-60.0 | 14.3-55.6 |
|  |  | (n) | (119) | (85) | (104) | (51) | (59) |
| *ACT: artemisinin-based combination therapy; AMT: artemisinin monotherapy; nAT: non artemisinin therapy; N: total number of retail outlets from which pricing data was obtained; n: total number of individual antimalarial products audited contributing to the calculation of the weighted median; N/S: result not shown due to insufficient observations (n<5) to obtain a reliable estimate. 1: Private health facilities include both for-profit and not-for-profit facilities; Other private outlets include supermarkets, kiosks, itinerant medicine sellers (hawkers) and outlet types that do not fit into any of the mentioned outlet categories. Note: Because of missing data, mark-ups could not be calculated for 68.3% of audited products in Benin, 17.2% in Cambodia, 17.3% in the DRC, 33.9% in Nigeria, 16.6% in Uganda and 30.9% in Zambia.* | | | | | | | |
